# Using Health Information Technology to Engage African American Women on Nutrition and Supplement Use During the Preconception Period

**DOI:** 10.3389/fendo.2020.571705

**Published:** 2021-01-19

**Authors:** Paula Gardiner, Timothy Bickmore, Leanne Yinusa-Nyahkoon, Matthew Reichert, Clevanne Julce, Nireesha Sidduri, Jessica Martin-Howard, Elisabeth Woodhams, Jumana Aryan, Zhe Zhang, Juan Fernandez, Mark Loafman, Jayakanth Srinivasan, Howard Cabral, Brian W. Jack

**Affiliations:** ^1^Department of Family Medicine, University of Massachusetts Medical School, Worcester, MA, United States; ^2^Khoury College of Computer Sciences, Northeastern University, Boston, MA, United States; ^3^College of Health and Rehabilitation Sciences: Sargent College, Boston University, Boston, MA, United States; ^4^Department of Government, Harvard University, Cambridge, MA, United States; ^5^Department of Family Medicine, Boston University School of Medicine and Boston Medical Center, Boston, MA, United States; ^6^Institute for Health Systems Innovation and Policy, Boston University, Boston, MA, United States; ^7^Department of Obstetrics and Gynecology, Boston University School of Medicine and Boston Medical Center, Boston, MA, United States; ^8^Department of Family Medicine, Cook County Health System, Chicago, IL, United States; ^9^Department of Information Systems, Questrom School of Business, Boston, MA, United States; ^10^Department of Biostatistics, Boston University School of Public Health, Boston, MA, United States

**Keywords:** health information technology, health disparities, preconception care, supplement use, diet, nutrition

## Abstract

**Importance:**

Healthy nutrition and appropriate supplementation during preconception have important implications for the health of the mother and newborn. The best way to deliver preconception care to address health risks related to nutrition is unknown.

**Methods:**

We conducted a secondary analysis of data from a randomized controlled trial designed to study the impact of conversational agent technology in 13 domains of preconception care among 528 non-pregnant African American and Black women. This analysis is restricted to those 480 women who reported at least one of the ten risks related to nutrition and dietary supplement use.

**Interventions:**

An online conversational agent, called “Gabby”, assesses health risks and delivers 12 months of tailored dialogue for over 100 preconception health risks, including ten nutrition and supplement risks, using behavioral change techniques like shared decision making and motivational interviewing. The control group received a letter listing their preconception risks and encouraging them to talk to a health care provider.

**Results:**

After 6 months, women using Gabby (a) reported progressing forward on the stage of change scale for, on average, 52.9% (SD, 35.1%) of nutrition and supplement risks compared to 42.9% (SD, 35.4) in the control group (IRR 1.22, 95% CI 1.03–1.45, P = 0.019); and (b) reported achieving the action and maintenance stage of change for, on average, 52.8% (SD 37.1) of the nutrition and supplement risks compared to 42.8% (SD, 37.9) in the control group (IRR 1.26, 96% CI 1.08–1.48, P = 0.004). For subjects beginning the study at the contemplation stage of change, intervention subjects reported progressing forward on the stage of change scale for 75.0% (SD, 36.3%) of their health risks compared to 52.1% (SD, 47.1%) in the control group (P = 0.006).

**Conclusion:**

The scalability of Gabby has the potential to improve women’s nutritional health as an adjunct to clinical care or at the population health level. Further studies are needed to determine if improving nutrition and supplement risks can impact clinical outcomes including optimization of weight.

**Clinical Trial Registration:**

ClinicalTrials.gov, identifier NCT01827215.

## Introduction

Healthy nutrition during preconception has significant implications for the health of the mother and newborn. Maternal nutrition and health status at the time of conception is an important determinant of embryonic and fetal growth, and emerging evidence suggests that a mother’s diet and lifestyle influences the long-term health of her infant (1). Nutritional status is affected by numerous variables including access to healthy food choices and dietary supplements (e.g., folic acid), income, community environment, lifestyle habits such as smoking and participating in physical activity, and the presence of physiological stressors (2).

A healthy diet up to three years prior to conception reduces the risk of poor pregnancy and birth outcomes, including gestational diabetes, hypertensive disorders of pregnancy, and preterm birth. Evidence-based clinical guidelines include nutrition recommendations such as daily folic acid, appropriate iron and calcium consumption, healthy food choices, a safe amount of fish, and caffeine reduction to improve birth outcomes (3–5). However, healthcare providers often lack enough time and resources to deliver individualized care to reproductive-aged women with nutritional risks during a clinical encounter (6, 7).

African American and Black women are at increased risk of poor maternal health outcomes, including risks impacted by nutrition and health status. African American and Black women are likely to seek health information predominately from informal sources, such as social media and members of their social network (8, 9). Health disparities, longstanding institutional and structural barriers, and mistrust or lack of cultural awareness during the clinical encounter may contribute to the patient-provider communication gap. Additionally, little research has been conducted assessing multi-behavioral change related to nutrition among African American and Black women of reproductive age (10–12).

Health information technology may offer a promising and cost-effective way to introduce tailored healthy nutrition and dietary habits to women. To date, many e-health interventions are resource-intensive, include limited evidence-based information, or do not address underlying motivational factors that may affect behavior change (2, 5). Although interventions providing online education and counseling through the utilization of social media and text messaging show benefits among postpartum women, these interventions are much less effective among prenatal women (13) with limited studies showing the impact on preconception women. Due to limitations in providers being able to deliver appropriate health behaviors and risk reduction education during the clinical encounter, it is appropriate to study the use of innovative health technologies to address this gap. Conversational agents, computer-generated characters that simulate face-to-face conversation and communicate key health messages, can overcome barriers to the provision of health education and counseling in a variety of clinical areas (14, 15).

“Gabby” is an animated, conversational agent developed to bridge the gap between provider and patient. Informed by focus groups from the study population, Gabby presents as an African American female character who delivers culturally relevant preconception health education for over 100 risks across 13 domains, including nutrition and dietary supplement use (14, 16). Relevant risks identified through a baseline risk assessment are addressed using tailored dialogue incorporating behavioral change techniques like motivational interviewing and shared decision making. Behaviors requiring incremental, longitudinal change are facilitated through goal setting, positive reinforcement, problem-solving, tips, and homework. Sustained engagement is monitored through self-reported behavior change over a 12 month period (15, 17).

Progress in addressing health risks is assessed using the Transtheoretical Model of health behavior change (18). According to the model, there are five “stages of change” in the behavior change process. The Gabby system helps women progress from not thinking about making any changes in specific health behaviors (“precontemplation”) to actively changing or sustaining a behavior (“action” or “maintenance”) through stage-appropriate behavior change techniques.

We previously reported the results a randomized controlled trial (RCT) of 528 African American and Black women ages 18–34 designed to evaluate the effect of using Gabby that demonstrated a significantly greater increase in preconception risks progressing forward through the stages of the Transtheoretical Model (19) at 6 and 12 months compared to a control group when all 13 domains of preconception care were analyzed (20).

In this paper, we report a secondary analysis of data from this RCT to study the impact of Gabby on a subset of 480 women who reported at least one of the ten preconception health risks in the nutrition domain. The primary outcome “progressing forward” or “achieving action or maintenance” is the self-reported stage of change on each of the ten risks in the nutrition domain at 6 and 12 months.

## Materials and Methods

### Recruitment of Research Subjects, Baseline Data, and Randomization

Participants were recruited using research websites, community programs, and referrals. Those meeting inclusion criteria (self-reported African American or Black, ages 18–34, currently not pregnant) consented and baseline data was collected. Subjects were then randomized to intervention (Gabby) or control group. The manuscript describing the primary RCT contains details of the recruitment, enrollment, consent and randomization processes (20).

### Data Collection

Research staff collected contact information, socio-demographic characteristics including age (years), Hispanic, Latino or Spanish origin (yes/no), English as a primary language (yes/no), student status (yes/no), full or part-time employment (yes/no), household income (<$20,000, $20,000-49,999, >50,000), education (less than college, some college), marital status (single, never married, single with a partner, married, separated/divorced or widowed), how frequently they use the internet (occasionally, frequently, very frequently) and BMI (Body Mass Index) > 30.

### Risk Assessment

Following consent and baseline data collection, subjects were emailed their login information and asked to complete a 15 minute-risk assessment organized into 13 domains of preconception care (20). This paper focuses on the nutrition and supplement domain. **Table 1** lists the 10 nutrition and supplement risks, criteria for identification, and corresponding goal behavior.

**Table 1:** Nutrition and supplement risks, identification criteria, and goal behavior.

Subdomain	Preconception Care Risks	Criteria for Identification	Goal Behavior
**Food Choices**	Unhealthy Diet (<5 fruits/vegetables and junk food)	Response of < 5 daily servings of fruits & vegetables OR “Yes” in response to: Do you tend to snack on junk food (chips, soda, candy, desserts) most days?	Eating a healthier diet (<5 servings of fruits and vegetables a day and/or less junk food)
	>2 Servings of Fish	“Yes” to question: Do you eat fish more than twice a week? (Certain types of fish may have high levels of mercury, which could cause health problems)	Talking to a doctor about limiting the amount of fish you eat to the safe amount
	Low Omega-3 Fatty Acids in Diet	Indicating no intake of walnuts, olive oil or fatty fish	Eating more foods with Omega-3 Fatty Acids
**Supplements Needed for Preconception Care**	No Multivitamin with Folic Acid or Folic Acid Supplement	Selection of “None,” “Don’t Know,” or “Foods with folic acid” (without also selecting “Folic Acid Pill” or “Multivitamin with Folic Acid”) in response to: What way(s) do you get folic acid?	Taking a multivitamin or folic acid pill daily
	No Calcium	Not selecting “Calcium” in response to: “Do you take any of the following vitamins or minerals?	Getting more calcium
	No Iron	Not selecting “Iron” in response to: “Do you take any of the following vitamins or minerals?”	Getting more iron
	No Vitamin D	Not selecting “Vitamin D” in response to: “Do you take any of the following vitamins or minerals?”	Getting more vitamin D
**Supplements and Chemicals at Safe Consumption**	Vitamin A Supplement Use	Selection of Vitamin A in response to: “Do you take any of the following vitamins or minerals?”	Talking to a doctor about getting safe amounts of vitamin A
	Other Supplement Use	Have you ever taken herbs, home remedies, or weight loss products for your health?	Talking to a doctor about your supplements
	Caffeine Use	“Yes” to question: Do you drink caffeinated drinks like coffee, tea, soda or energy drinks?	Reducing to safe amount of caffeine (<200 mg a day)

### Intervention

After completing the risk assessment, subjects randomized to the Gabby intervention were asked to log on at least once every two weeks. Based on the relevant risks identified during the risk assessment, Gabby compiles a customizable list called the “My Health To-Do List” from which a woman can choose to discuss health topics that Gabby tracks over time.

Through unfolding conversational dialogue, Gabby addresses the following topics: adequate intake of fruits and vegetables, unhealthy food choices (e.g., junk food and sweets), the risk for mercury consumption when eating certain kinds of fish, omega-3 fatty acids rich food consumption, multivitamin with folic acid or folic acid supplement, calcium-rich food or supplement consumption, iron-rich food or supplement consumption, vitamin D rich food or supplement consumption, vitamin A overuse, use of herbal or weight loss supplements, and caffeine consumption. The content programmed in the Gabby system to mitigate these risks is informed by the AJOG evidence-base on nutrition and dietary supplements (5), CDC diabetes prevention curriculum (21), Five Fruit and Vegetables recommendation (22), Harvard School of Public Health’s healthy eating plate (23) and messaging based on key health behavior techniques (24–27).

The scripted dialogues Gabby delivers for healthy eating encompasses the following modules: fruits and vegetables, healthy eating plate (23), fat awareness, portion sizes, and the relationship between stress and eating. The content surrounding vitamin and mineral-rich food or supplement needs include folic acid, multivitamins, calcium, iron, vitamin D, vitamin A, herbal supplements, and omega-3 fatty acids. Gabby also provides dialogue on maintaining a healthy weight, setting goals about nutrition and supplement use, limiting caffeine intake, and references a variety of food choices that align with the range of dietary norms and preferences among subjects.

**Figure 1:**
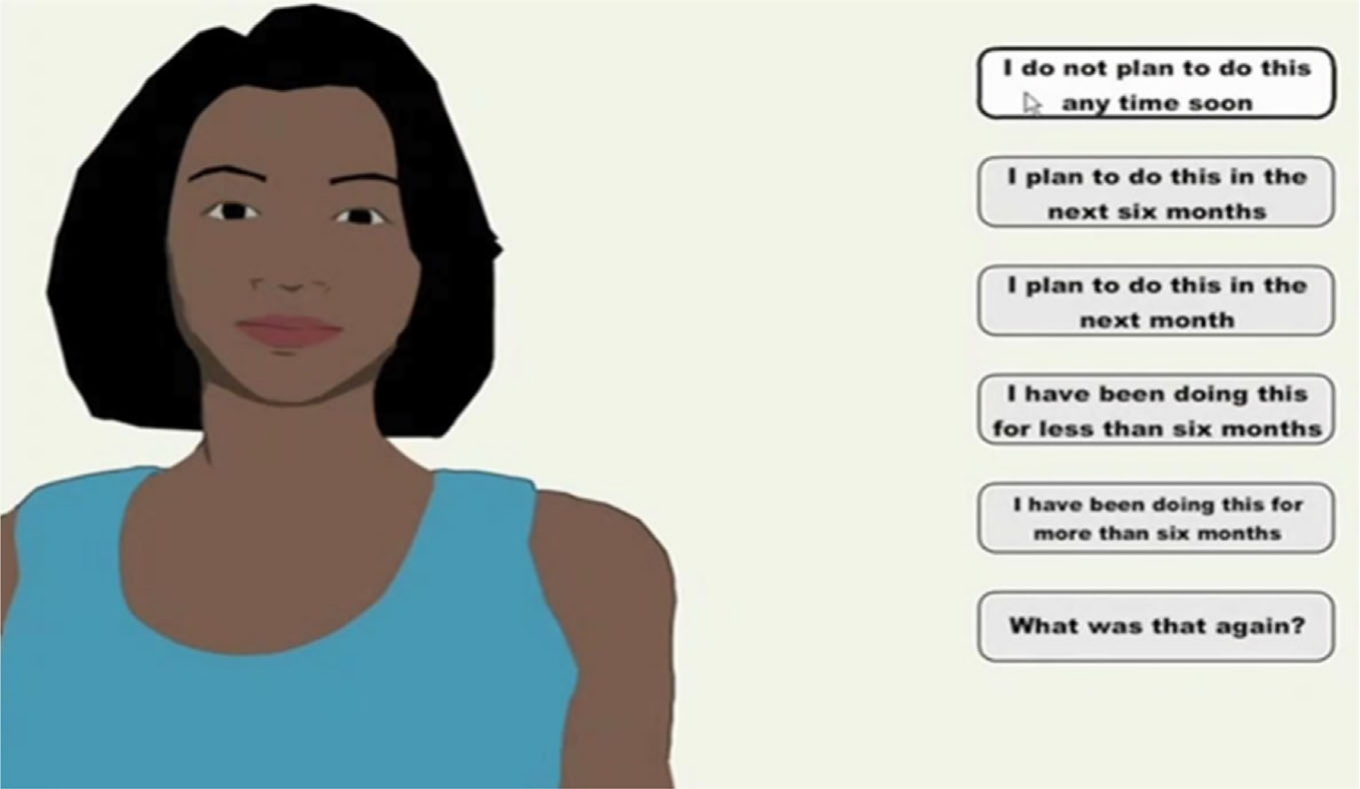
Image of Gabby, the preconception care conversational agent, with response buttons to facilitate dialogue.

Gabby then describes why managing any risk identified is important and offers suggestions about how to take action on it. For example, Gabby will provide tips on finding low-calorie substitutions for meals, suggest healthier ways to cook meat (i.e., grilling, broiling, charring) and vegetables (i.e., steaming, boiling, microwaving), and draining fat by blotting with a paper towel. During up to 12 months of subsequent interactive dialogue, Gabby identifies the degree of progress, and provides feedback on actions taken, reassesses readiness, and recommends next steps. The system dynamically assesses the woman’s level of understanding of health information provided and repeats information when requested by the subject. Gabby uses nonverbal conversational behaviors such as hand gestures and facial expressions, while the woman responds by selecting a response from a multiple-choice menu of options updated at each turn of dialogue as depicted in **Figure 1**.

### Control Group

Controls received a letter that listed the health risks reported on their risk assessment and encouraged women to discuss these risks with a health care professional.

### Data Collection at 6 and 12 Months

At baseline, 6, and 12 months, all subjects were asked by telephone interview to report their stage of change for addressing each of the risks that were identified on the baseline risk assessment.

### Primary Outcomes

Self-reported stage of change data was collected and used to construct two primary outcomes: the self-reported percentage of preconception care risks from the nutrition domain that (1) progressed forward on the stage of change scale, and (2) reached the action or maintenance stage of change.

### Secondary Outcomes

Secondary outcomes include (1) total usage of the entire system for all risks (logons and minutes), (2) the percentage of risks progressing forward and reaching action or maintenance within three subdomains within the nutrition domain, and (3) the odds of each of the individual ten nutrition risks progressing forward and reaching action or maintenance. The three subdomains constructed were: food choices, including unhealthy diet, greater than 2 servings of fish, and low omega-3 fatty acids; supplements needed for preconception care, including folic acid, iron, calcium, and vitamin D supplementation; and supplements and chemicals to use at a safe level, including safe vitamin A use, other supplement use, and caffeine use.

### *A Priori* Subgroup Analyses

Subgroup analyses explored whether the relationship between nutrition risks and the primary outcome was differentially effective among subgroups of clinical and demographic variables. Variables analyzed include education, income, age, and nutrition knowledge, which was a 6-item assessment on folic acid dose, types of folic acid in diet, knowledge of supplements for bones (calcium), blood (iron), healthy fats, and number of recommend fruits and vegetables. The assessment was scored on a range of 0-6 and rescaled as a percent of correct responses.

### Statistical Analysis

Balance across study arms at baseline was assessed using independent sample t-tests for continuous variables and chi-squared tests for dichotomous variables. All treatment effects were reproduced using regression to control for covariates unbalanced at baseline.

Primary outcomes were assessed in two ways. First, we used intention-to-treat (ITT) analysis to detect whether the Gabby intervention exerted an effect on each of our two primary outcomes, at both 6 and 12 months, across all participants that were randomized. These analyses were conducted by transforming percentages into rates and regressing those rates on both study arms using Poisson regressions.

We then conducted an as-treated analysis by regressing the primary outcomes, at 12 months, on the number of logins to the Gabby system over those 12 months, using Poisson regression, and including all participants that logged in to the Gabby system at least once. In this analysis, we determined *a priori* that three potential confounding variables (education, income, and age) may correlate with progress on the stage of change and Gabby system usage, and controlled for them in the regression analysis.

For secondary outcome analyses, for each subdomain, the data was restricted only to subjects who flagged at least one risk within that subdomain, and Poisson models were used to regress the rate of progressing forward or achieving action or maintenance on both study arms. In addition, a logistic regression model was used to regress a binary measure, for each risk, of whether that risk had either progressed forward or remained at action or maintenance on study arm. Poisson and logit coefficients were used to generate odds and rate ratios with 90% and 95% confidence intervals.

*A priori* subgroup analyses were conducted across both study arms by using Poisson models to regress the rate of nutrition and supplement risks that progressed forward or reached action or maintenance on subgroup variables: education, income, age, and nutrition knowledge. Analyses were also conducted, with Poisson models, to determine the differential effects of Gabby across subgroups by regressing the rate of nutrition and supplement risks that progressed forward or reached action or maintenance on both study arms interacted with these subgroup variables.

An analysis was conducted to determine whether Gabby was differentially effective conditional on the stage of change from which they started at baseline. This was done by restricting the data only to risks that were reported at “precontemplation” at baseline; then by calculating the rate that progressed forward or remained at “action” or “maintenance,” and finally by regressing that rate on both study arms using a Poisson model. This procedure was repeated by restricting the data to risks reported at “contemplation” at baseline, and again by restricting to risks reported at the “preparation” and then “action” stages of change.

All statistical tests were run at alpha level a=0.05 and were performed using the R programming language, version 3.4.3.

## Results

### Recruitment and Enrollment

Among the 528 subjects recruited in the full randomized trial, 480 subjects flagged at least one of the 10 risks related to nutrition and supplement use. Although randomization was not blocked on the presence of a nutrition risk, by chance there were 240 women in both the intervention and control groups who flagged a nutrition risk. Complete outcome data were obtained for 116 intervention and 146 control subjects at 6 months, and 105 intervention and 124 control subjects at 12 months – no significant differences between study arms were detected on baseline covariates (age, Hispanic origin, language, education, employment, income, marital status) amongst subjects missing 6- or 12-month data.

### Baseline Data Collection

**Table 2** shows the sociodemographic and clinical characteristics of the 480 women studied. Participants averaged 27 years old, 89 percent were either a student or worked full or part-time, and 92 percent attended at least some post-secondary school education. There were differences between study arms in the number of subjects identifying English as their primary language (P = 0.024), and the number of subjects accessing a computer primarily from home (P = 0.046) and work (P = 0.038). All subsequent ITT analyses were checked by controlling for these three unbalanced variables.

**Table 2:** Sociodemographic and clinical characteristics across study arms.

	*Gabby Group (n = 240)*	*Control Group (n = 240)*
***Demographics***		
***Age, mean years (SD)***	27 (3.91)	27 (4.39)
***Hispanic, Latino or Spanish Origin***	11 (4.6)	12 (5.0)
***English as Primary Language***	229 (95.4)	238 (99.2)
***Currently a Student***	54 (22.5)	67 (27.9)
***Currently Employed Full or Part Time***	190 (79.2)	190 (79.2)
***Household Income ($)***		
*Less than $20,000*	17 (7.1)	17 (7.1)
*$20,000 - 49,999*	83 (34.6)	77 (32.1)
*$50,000 or more*	110 (45.8)	113 (47.1)
***Education***		
*At Least Some College*	218 (90.8)	223 (92.9)
*Less than College*	22 (9.2)	17 (7.1)
***Marital Status***		
*Single, never married*	154 (64.2)	148 (61.7)
*Single, with partner*	41 (17.1)	52 (21.7)
*Married*	37 (15.4)	37 (15.4)
*Separated, divorced or widowed*	8 (3.3)	3 (1.2)
***How Frequently Do You Use the Internet for Health Information?***		
*Occasionally*	83 (34.6)	100 (41.7)
*Frequently*	78 (32.5)	71 (29.6)
*Very Frequently*	59 (24.6)	54 (22.5)
***Clinical Characteristics***		
***BMI > 30***	96 (40)	103 (42.9)

Data are n (%) unless otherwise indicated.

Data do not add up to 100% due to missing data. BMI, Body Mass Index.

### Preconception Risk Assessment

**Table 3** shows the nutrition and supplement risks and the stage of change for each at baseline, 6 months, and 12 months. Of those subjects flagging a nutrition and supplement risk, 77.9% of subjects flagged the unhealthy diet risk behavior, 27.9% flagged >2 servings of fish, 25.2% flagged need omega-3 fatty acids, 62.5% flagged no folic acid supplement, 74.0% flagged no iron supplement, 62.1% flagged no calcium supplement, 50.0% flagged no vitamin D supplement, 19.2% flagged vitamin A supplement use, 61.0% flagged other supplement use, and 76.0% flagged caffeine. Both intervention and control subjects flagged an average of five out of 10 possible nutrition and supplement risks (P = 0.203). The number of risks flagged ranged from 4 at the bottom quartile to 7 at the top quartile.

**Table 3:** Nutrition and supplement risks and mean stage of change (soc) values at baseline, 6 and 12 months.

Preconception Care Risks in 3 Subdomains	Baseline		6 Months		12 Months	
	Mean SOC, Control Group (N)	Mean SOC, Gabby Group (N)	Mean SOC, Control Group (N)	Mean SOC, Gabby Group (N)	Mean SOC, Control Group (N)	Mean SOC, Gabby Group (N)
Unhealthy Diet (<5 fruits/vegetables/junk food)	3.05 (167)	3.09 (172)	3.50 (116)	3.84 (96)	3.73 (104)	3.97 (90)
>2 Servings of Fish	1.89 (46)	2.26 (47)	1.50 (24)	2.67 (24)	2.24 (21)	2.65 (23)
No Omega-3 Fatty Acids	2.76 (50)	2.83 (53)	2.89 (35)	3.20 (30)	3.38 (24)	3.56 (27)
**Food Choices**	**2.79 (190)**	**2.90 (189)**	**3.10 (130)**	**3.53 (108)**	**3.46 (115)**	**3.67 (101)**
No Folic Acid Supplement	2.35 (127)	2.39 (140)	3.13 (89)	3.29 (85)	3.32 (74)	3.45 (71)
No Calcium	2.79 (121)	2.58 (134)	3.12 (82)	3.32 (97)	3.33 (69)	3.45 (67)
No Iron	2.72 (152)	2.55 (142)	3.32 (97)	3.46 (83)	3.29 (85)	3.58 (72)
No Vitamin D	2.62 (100)	2.47 (107)	3.20 (64)	3.34 (61)	3.31 (61)	3.67 (45)
**Supplements Needed**	**2.62 (174)**	**2.5 (177)**	**3.12 (120)**	**3.35 (108)**	**3.31 (101)**	**3.53 (92)**
Vitamin A Supplement Use	2.21 (38)	2.32 (25)	2.32 (28)	2.50 (20)	2.21 (24)	2.79 (19)
Other Supplement Use	2.08 (111)	2.29 (108)	2.40 (57)	2.71 (55)	2.43 (58)	2.96 (51)
Caffeine Use	2.98 (134)	2.93 (123)	3.27 (77)	3.53 (66)	3.39 (71)	3.55 (51)
**Supplements and Chemicals at Safe Consumption**	**2.52 (189)**	**2.60 (168)**	**2.80 (113)**	**3.06 (95)**	**2.84 (106)**	**3.18 (81)**

### 6 and 12 Month Outcomes

Although this analysis was not powered to detect a treatment effect on nutrition and supplement risks alone, nevertheless we were able to detect an effect of Gabby at 6 months. As shown in **Table 4**, at 6 months, women assigned to use Gabby reported moving forward on the stage of change scale for, on average, 52.9% (SD, 35.1%) of the nutrition and supplement risks flagged compared to 42.9% (SD, 35.4%) in the control group (IRR 1.22, 95% CI 1.03–1.45; P = 0.019). Similarly, women assigned to use Gabby reported reaching the action or maintenance stage of change for, on average, 52.8% (SD, 37.1%) of the nutrition and supplement risks flagged compared to 42.8% (SD, 37.9%) in the control group (IRR 1.26, 95% CI 1.08–1.48; P = 0.004).

**Table 4:** Stage of change outcomes for diet and nutrition preconception risks at 6 and 12 months by intervention and control groups.

	Control Group (n = 240) reported % *preconception risks (SD)^†^	Intervention Group (n = 240) reported %preconceptionrisks (SD)	Rate Ratio^||^(95% CI)	P-Value^‡^
**At 6 months**				
Risks that progressed^§^	42.9% (35.4)	52.9% (35.1)	1.22 (1.03, 1.45)	0.019
Risks at action or maintenance^¶^	42.8% (37.89)	52.8% (37.14)	1.26 (1.08, 1.48)	0.004
**At 12 months**				
Risks that progressed	49.8% (36.61)	54.3% (38.72)	1.01 (0.85, 1.20)	0.928
Risks at action or maintenance	53.9% (38.06)	61.8% (37.70)	1.12 (0.95, 1.31)	0.168

*Percentage of behaviors (at action, at maintenance, progressed) is the rate per 100 behaviors triggered.

^†^Standard deviation of the percentage of behaviors.

^||^Gabby group rate divided by the usual care group rate.

^‡^From a Poisson regression of the rate on study arm.

^§^On the ordered stage of change scale, pre-contemplation < contemplation < preparation < action < maintenance.

^¶^On the stage of change scale (pre-contemplation, contemplation, preparation, action, maintenance); only some behaviors were assessed with a maintenance stage.

At 12 months, women interacting with Gabby, on average, reported making forward progress on the stage of change scale for 54.3% (SD, 38.7%) of nutrition and supplement risks compared to 49.8% (SD, 36.6%) in the control group (IRR 1.01, 95% CI 0.85–1.20; P=0.928). Similarly, women assigned to use Gabby, at 12 months, reported reaching the action or maintenance stage of change for 61.8% (SD, 37.7%) of nutrition and supplement risks compared to 53.9% (SD, 38.1%) in the control group (IRR 1.12, 95% CI 0.95–1.31; P = 0.168).

All treatment effects at 6 months remained statistically significant after controlling for primary language, which was unbalanced at baseline.

Among women using Gabby, the behaviors most often progressing forward on the stage of change scale were unhealthy diet, no folic acid supplement, and no iron supplement; these were also the behaviors most often reaching the action or maintenance stages.

### As-Treated Analysis

Women who used Gabby more often were more effective at addressing their nutrition and supplement risks. When analyzing only those who used Gabby, each additional login was associated with an average increase of 1.01% in the reported rate of achieving the action or maintenance stage or progressing forward at 12 months (P = 0.014). This result remained significant when controlling for education, income, and age. For example, for a woman logging on to Gabby 10 times over the study period, to achieve the outcome for one additional risk, that woman would need to log on an additional 2.4 times per month, on average.

### Secondary Outcomes

198 of the 240 women enrolled in the intervention group interacted with the entire Gabby system at least once, and the median number of logins was 6 (IQR 8) after 12 months. The median duration of a session with Gabby was 13.7 min (IQR 8.7 min). The median average interaction time (not including the risk assessment) with Gabby was 73.8 min (IQR, 134.6) per woman who used Gabby. The aforementioned data was derived from usage of the entire Gabby system (20); We are unable to report the unique usage data specific to the nutrition and supplement domain.

**Figure 2:**
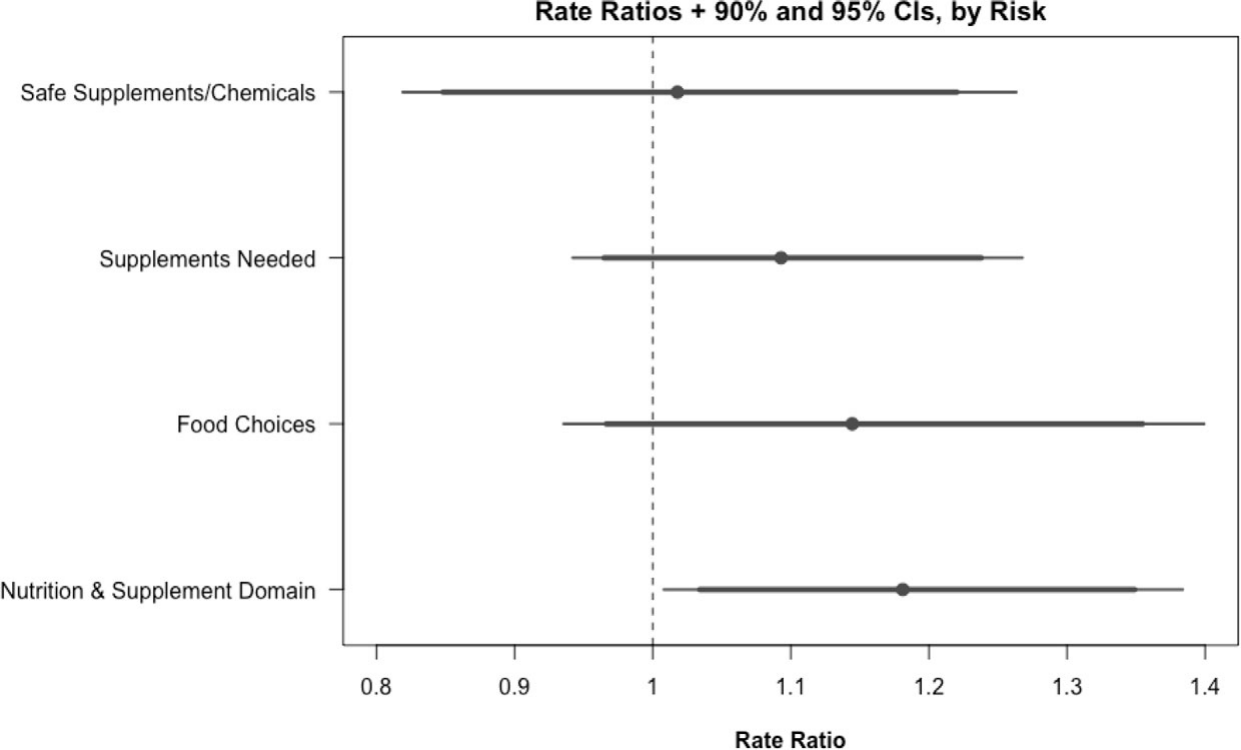
Rate ratios and 90%/95% CIs, by subdomain. Rate ratios and confidence intervals are calculated by regressing the rate of each risk achieving action or maintenance or making progress on the stage of change scale on each study arm using logistic regression.

**Figure 2** plots rate ratios with 90% and 95% confidence intervals and across three subdomains of nutrition and supplement risk behaviors and the entire domain. Rate ratios were calculated for the rate of either progressing forward or achieving action or maintenance on the stage of change scale. Because fewer subjects flagged risks within a subdomain than within the broader nutrition and supplement domain, sample sizes for within-subdomain effects are small. Nevertheless, several within-subdomain differences across study arms were of notable magnitude. Within the food choices subdomain, at 6 months, on average, subjects in the intervention group reported reaching the action or maintenance stage of change or progressing forward on the stage of change scale for 11.23% more risk behaviors than subjects in the control group (62.76% versus 49.17%, P = 0.165); at 12 months, the difference was 10.96% (73.33% versus 62.38%, P = 0.401). Similar results were found for the supplements needed subdomain (65.02% versus 58.33% at 6 months, P = 0.219).

**Figure 3:**
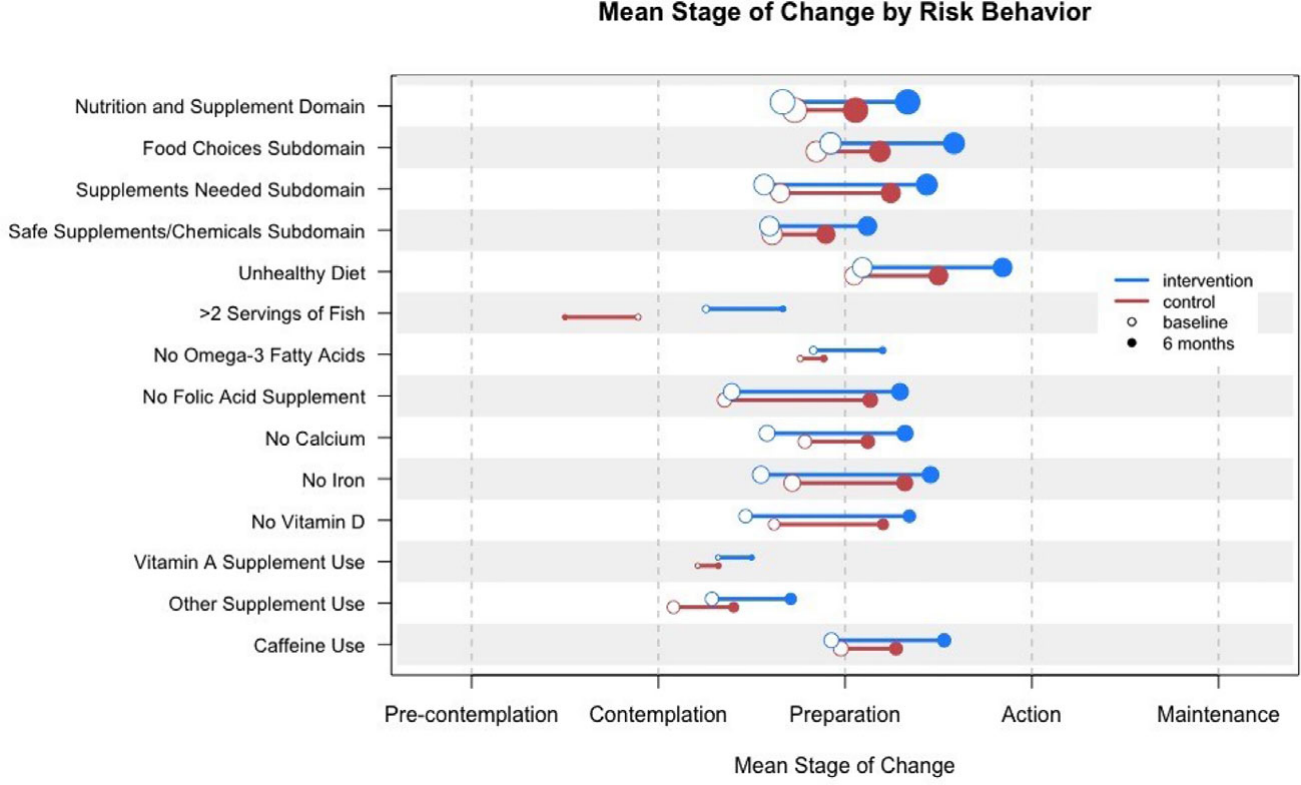
Mean stage of change for nutrition and supplement domain, subdomain, and individual risks at baseline and 12 months for the intervention and control groups. Circle size indicates the relative number of women in the sample who triggered the risk.

**Figure 3** shows the mean stage of change for the nutrition and supplement domain, subdomain, and individual risks at baseline, at 6 and 12 months for the intervention and control groups. Subjects assigned to the intervention group performed no worse than subjects assigned to the control group on any single risk behavior. For one risk behavior (i.e., having more than two servings of certain types of fish per week) subjects assigned to the intervention group were more likely to achieve action or maintenance or make forward progress than subjects in the control group (OR 5.34, CI 1.42–22.9).

### Subgroup Analyses

Subgroup analysis also showed that the effect of Gabby on addressing nutrition and supplement risks was larger for subjects with higher income (P = 0.029), and for subjects with lower nutrition knowledge (P = 0.065). Gabby was equally effective at addressing nutrition and supplement risks across levels of education, income, and age.

**Figure 4:**
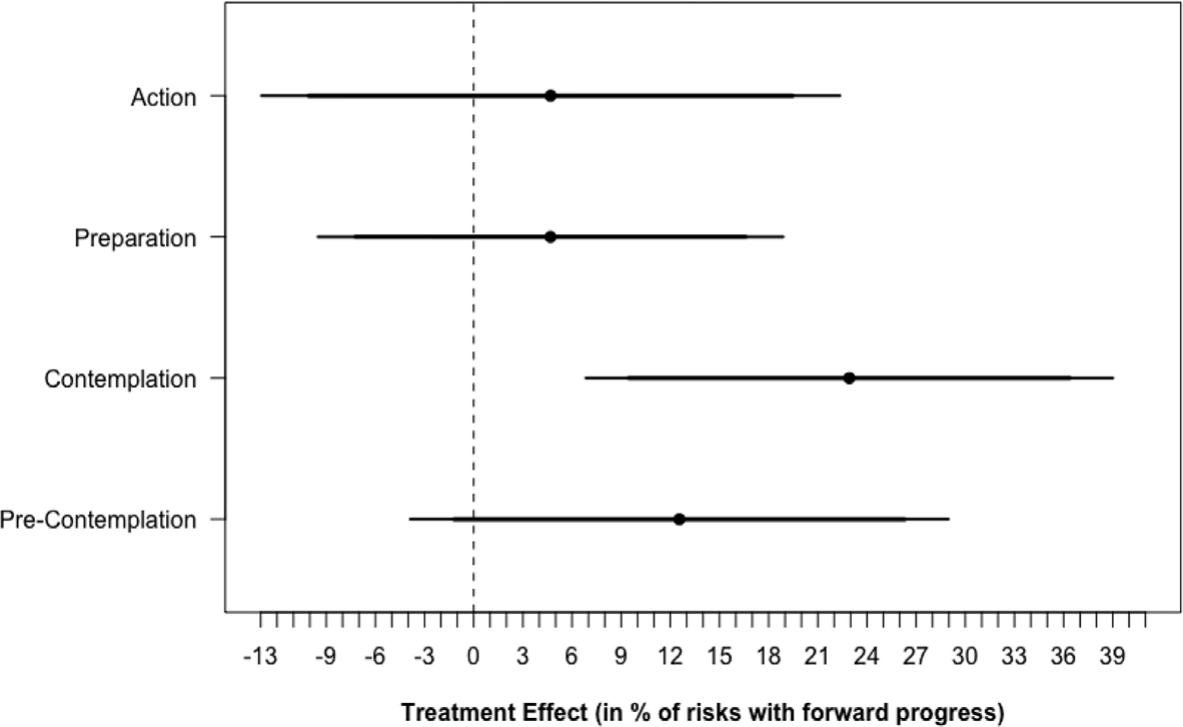
Treatment Effect* by risk stage of change at baseline. * Treatment effects confidence intervals are calculated by restricting the data only to subjects that began the study at each stage of change and regressing the percentage of risks making forward progress on the stage of change scale on study arm using OLS regression.

**Figure 4** shows that the Gabby intervention had a significant positive effect on the primary outcomes when restricting the data only to risks beginning the study at the contemplation stage of change. For subjects at the contemplation stage at baseline, intervention subjects reported making forward progress for 75.0% (SD, 36.3%) of their health risks compared to 52.1% (SD, 47.1%) in the control group (P < 0.056). Similar results were not found for subjects beginning at the precontemplation, preparation, or action stages of change. Differences in statistical significance across subjects beginning at different stages were unlikely due to differing degrees of freedom, but effects detected for subjects beginning the study at the precontemplation, preparation, and action phases were small in magnitude and may only be detectible with larger sample sizes and increased statistical power. Results were unchanged after controlling for level of education, income, age, and nutrition knowledge.

## Discussion

This secondary analysis shows that among women using Gabby, there was a 22% increase in the average number of preconception nutrition and supplement risks that women reported progressing forward on the stage of change scale at 6 months, and a 26% increase the number of rusks that reach the action or maintenance stages of change. The randomized trial from which these data were collected was powered to demonstrate an impact on our primary outcomes when 102 preconception risks in 13 domains of care were analyzed. The main finding of this study demonstrating the impact of the intervention in a single domain is surprising. The results are especially noteworthy given that the control group received a letter listing their nutrition and supplement risks and suggested they discuss their risks with a clinician, which is an intervention that could be potentially impactful on its own. Our data suggest that Gabby was more effective in addressing nutrition and supplement risks among women with lower nutrition knowledge but equally effective across levels of education, income, and age.

This study serves as one of the first to analyze the dietary habits and supplement use of African American and Black women of childbearing age. Interventions that aim to understand how to change underlying health behaviors that impact maternal and child health outcomes are critical in addressing poor health outcomes, particularly in health disparate populations (28). African American and Black women experience higher rates of unintended pregnancies, complications, and pregnancy-related and infant deaths due to barriers accessing health information and services (29), like gaps in nutritional knowledge and healthy food choices.

Other studies have assessed the impact of various eHealth platforms on nutrition, supplement use, and other preconception risks. Using a coaching platform, the Smarter Pregnancy study conducted in the Netherlands was designed to address the impact of an eHealth intervention on the behavior change of couples who were pregnant or considering pregnancy. After 6 months of usage, there was an increase in vegetable intake, tobacco cessation, and no alcohol consumption among study participants after receiving tips, recommendations, and recipes regarding pregnancy, BMI, and nutrition *via* text and email (30). Another eHealth study evaluated the SmartMoms intervention designed to manage gestational weight gain among overweight and obese pregnant women. While the in-person and remote groups experienced similar weight gain, the remote group had better adherence to the intervention than the in-person group, suggesting a benefit to mobile health interventions compared to traditional in-person interventions (31). Finally, PEARS, a smartphone application intervention that assessed diet and physical activity behavior change in overweight or obese pregnant women observed that after the intervention, the majority of participants using the application were in the maintenance stage for perceived physical activity (32).

Our data demonstrate that the more time women spent using Gabby, the more effective they were in addressing their nutrition and supplement risks. Gabby provides the opportunity for convenient counseling and advice, as our data show that the total average interaction time with Gabby was over 73 min – substantially longer than a routine annual office visit in many health care systems.

When beginning the study at the contemplation stage, subjects assigned to use Gabby reported making forward progress on 44% more risk behaviors than the control group, indicating that Gabby greatly assisted women who were already considering behavior change. However, despite our efforts to create motivational interviewing dialogue to assist women to progress from precontemplation to contemplation, we did not observe any effect of Gabby on addressing supplement and nutrition risks for this subset of women. No similar effects were detected for subjects beginning the study at the precontemplation, preparation, or action phase. While the study was not powered to detect this change, it could also be due to unsuccessful dialogue content.

This study has several limitations. As a secondary analysis of a randomized trial, the two arms were not blocked on nutrition and supplement risks during randomization. However, our analysis controlled for demographic and clinical variables that were unbalanced at baseline. Second, there were differences in the number of women reporting outcomes at 6 and 12 months. It could be that those women who did not provide outcome data at 6 months did so because they felt it to be not relevant to their current behaviors, though characteristics of women missing 6- and 12-month data were nevertheless balanced across study arms.

A second limitation to this study is that this analysis did not show that the impact on the primary outcomes was maintained at 12 months; however, the fully powered trial from which this secondary analysis is based on showed that the impact on the primary outcomes was maintained at 12 months. It is possible that the lack of maintaining behavior change is the result of an inadequate sample size.

Finally, we do not know the number of minutes that women spent specifically engaging with the nutrition and supplement domain so we cannot extrapolate whether our findings are due to interacting with the entire system or unique content.

Gabby can assist with health risks requiring long-term longitudinal behavior change such as improving nutrition and supplement use. It is an engaging system that provides tailored dialogue, including motivational interviewing and shared decision making, and monitors a woman’s progress over time – providing interaction necessary to address complex and challenging clinical problems like overweight and obesity.

A scalable health information technology tool like Gabby could undoubtedly be an important component of future health education, risk identification and mitigation. Gabby could be used as an adjunct to clinical care to overcome the barrier of clinician time restraints and could be particularly impactful as a population health tool. Gabby allows women to have evidence-based conversations about nutrition and dietary supplement use on their own time without restriction.

## Data Availability Statement

The raw data supporting the conclusions of this article will be made available by the authors, without undue reservation. Individual participant data will be available, including data dictionaries. Data that will be shared: Individual participant data that underlie the results reported in this article, after deidentification (text, tables, figures). 6 and 12 months Stage of Change, System Usage, Risk Assessment, Baseline Data Collection (Demographics and Clinical Characteristics). Study Protocol, Statistical Analysis Plan, Analytic Code (R). RedCap exported data will also be available. Data will be available immediately following publication for a period of 5 years. Data will be shared with researchers who provide a methodologically sound proposal to achieve aims related to primary/secondary outcomes. Proposals should be sent to bjack@bu.edu. To gain access, data requestors will need to sign a data access agreement.

## Ethics Statement

The studies involving human participants were reviewed and approved by the Boston University Institutional Review Board. A waiver of documentation of consent was approved and only verbal consent was obtained. As such written informed consent did not require the subjects signature for study participation in accordance with the national legislation and the institutional requirements.

## Author Contributions

BJ, PG, CJ, and NS wrote the first draft of the manuscript with input from LY-N, ML, JS, JM-H, and EW. CJ and JM-H conducted study activities. BJ, CJ, EW, and LY-N contributed to Gabby script development. JA contributed to the discussion section of this manuscript with current literature and trends. TB, ZZ, and JF created and programmed the Gabby system and added several features to the updated version. MR did the statistical analysis and wrote corresponding sections of the manuscript. HC oversaw statistical analyses and provided guidance into study-appropriate statistical measures. All authors contributed to the article and approved the submitted version.

## Funding

Research reported in this publication was supported by the National Institute for Minority Health and Health Disparities grant R01MD006213.

## Conflict of Interest

The authors declare that the research was conducted in the absence of any commercial or financial relationships that could be construed as a potential conflict of interest.
